# Supplementary material for: Microinjection to deliver protein, mRNA, and DNA into zygotes of the cnidarian endosymbiosis model Aiptasia sp
Source: Sci Rep. 2018 Nov 6;8:16437. doi: 10.1038/s41598-018-34773-1 (PMC6219564; doi:10.1038/s41598-018-34773-1)
Supplement: Supplementary file 3 — Supplementary Information [file 41598_2018_34773_MOESM3_ESM.pdf]

Supplementary Information

Microinjection to deliver protein, mRNA, and DNA into zygotes of the cnidarian endosymbiosis model *Aiptasia* sp.

Victor A. S. Jones, Madeline Bucher, Elizabeth A. Hambleton, Annika Guse

Figure S1

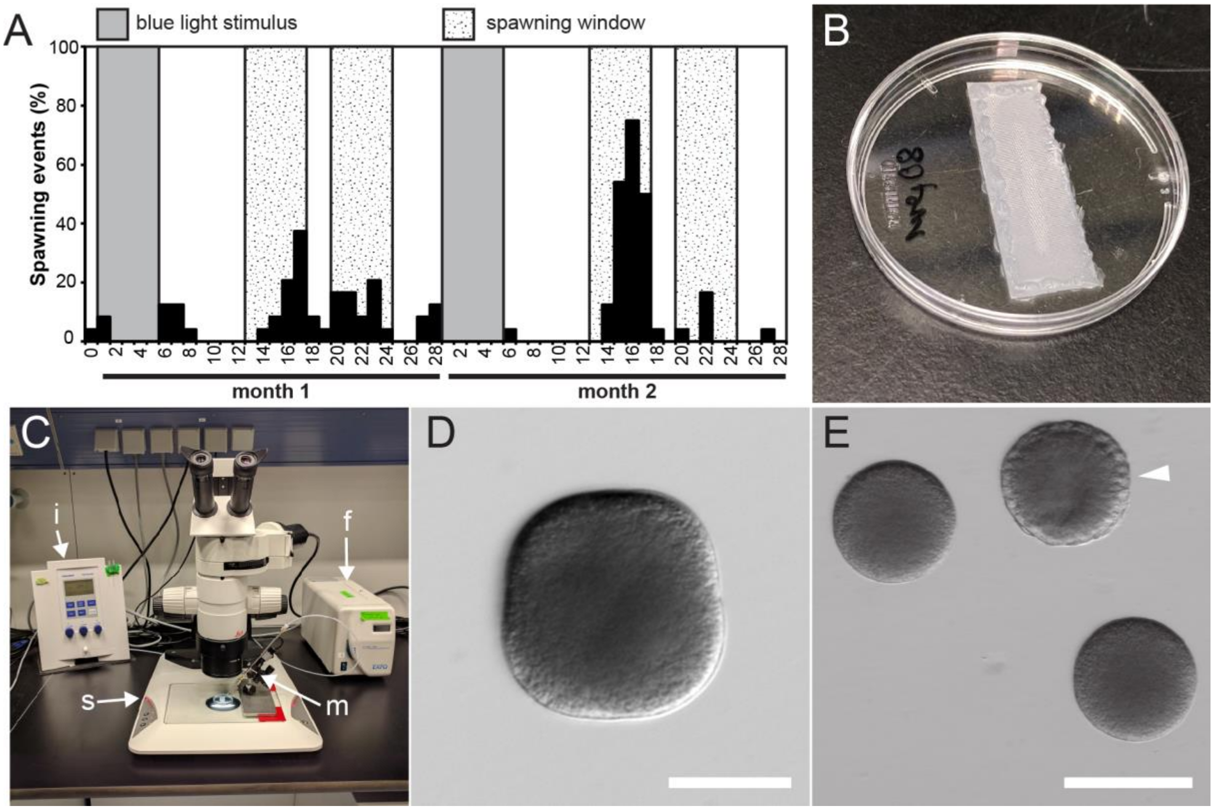

(**A**) Spawning induction efficiency is demonstrated by the percentage of female tanks containing spawned eggs on each day of two consecutive artificial lunar cycles. Duration of the blue-light stimulus (spawning induction cue) is indicated in grey; the expected spawning period is indicated with a speckled background (total of 24 tanks). (**B**) Microinjection dishes are prepared by affixing a strip of 80 x 80 µm nylon mesh on the bottom of a small petri dish lid using silicon grease around the edges. (**C**) Microinjection set-up with stereomicroscope (s), fluorescence lamp (f), micromanipulator (m), injection pedal (not shown), and FemtoJet 4i injector (i). (**D**) The injection session proceeds until most zygotes assume a box-like shape, indicating imminent cleavage to 4-cell stage. Scale = 100 µm. (**E**) Developing embryos (arrowhead) and unfertilized eggs can be clearly differentiated 4-5 h post-fertilization (hpf). Scale = 50 µm.

Figure S2

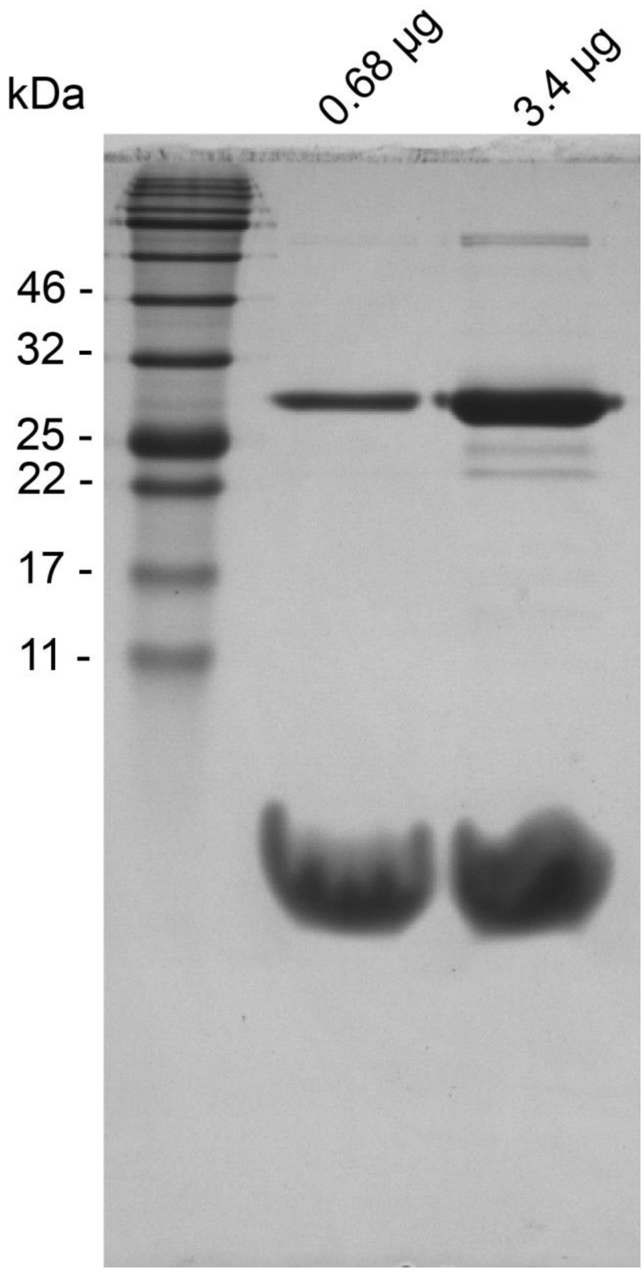

Recombinant Lifeact-eGFP protein (30.6 kDa) resolved with SDS-PAGE and stained with Coomassie Brilliant Blue to visualize. Protein was injected at 3.4 mg/ml into zygotes. Left lane, ladder.

Figure S3

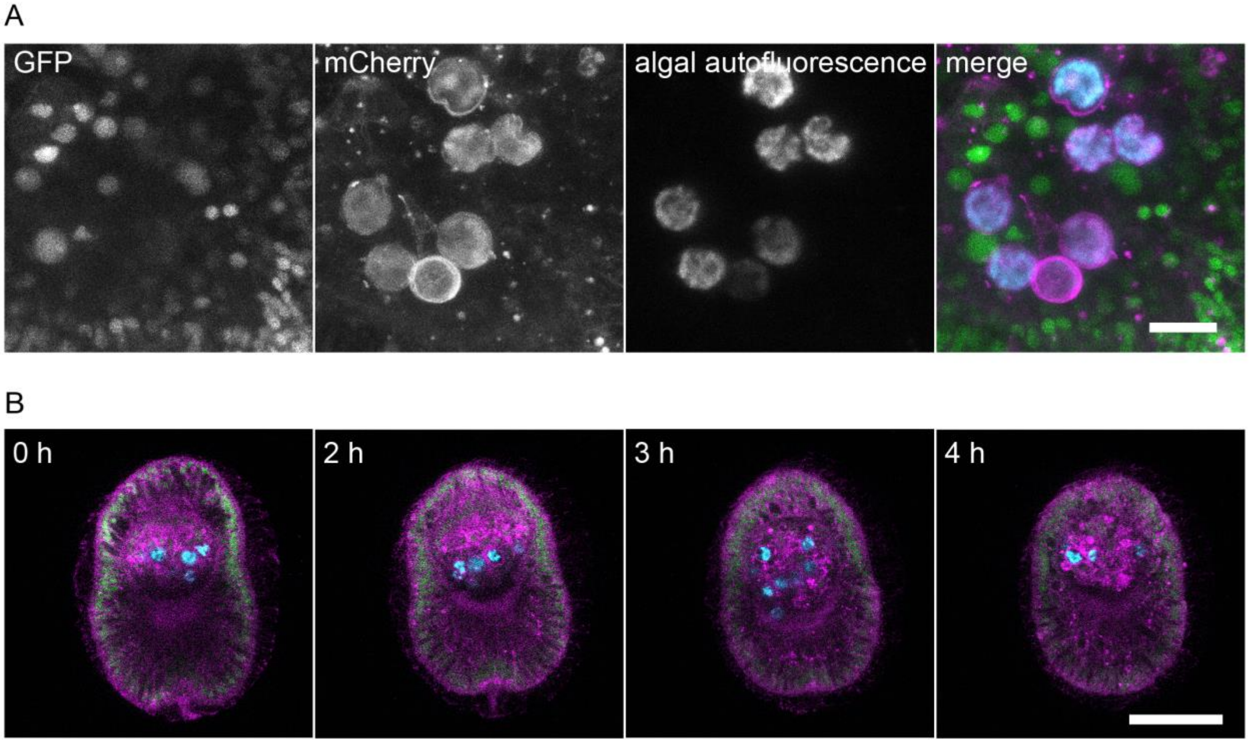

(**A**) Within a single *Aiptasia* larva, the strength of symbiosome labeling by farnesylated mCherry (magenta) and algal far-red autofluorescence (cyan) are variable. Larva was injected with *NLS-eGFP-2A-mCherry-CaaX* mRNA and incubated for 2 days with *Symbiodinium* strain SSB01 at 10,000 cells/ml, before fixation at 4 dpf. Scale = 10 µm. (**B**) Agarose-embedded larvae can be imaged live for several hours. Larva was injected with *NLS-eGFP-2A-mCherry-CaaX* mRNA and incubated for 1 day with *Symbiodinium* strain SSB01 at 10,000 cells/ml, then at 3 dpf embedded in low-melt agarose and imaged at the shown times. Z-stacks were taken with confocal laser-scanning microscopy once every 30 min. Scale = 50 µm.

Movie S1
Live imaging of larvae after incubation with *Symbiodinium* strain SSB01 at 100,000 cells/ml for 2 days. Larva was embedded in low-melt agarose and imaged at 4 dpf with DIC and fluorescence microscopy to monitor intracellular symbionts via their natural autofluorescence using a TexasRed filter set. False color representation with 5 ramps LUT. Scale = 100 µm.

Movie S2
Live imaging of the endoderm of a larva injected with *NLS-eGFP-2A-mCherry-CaaX* mRNA and incubated for 1 day with *Symbiodinium* strain SSB01 at 100,000 cells/ml. Larva was embedded in low-melt agarose and imaged at 3 dpf with confocal laser-scanning microscopy, at a rate of one frame per 2.5 sec. mCherry channel left, grayscale; merge of mCherry (magenta), eGFP (green) and algal red autofluorescence (cyan), right. Scale = 10 µm.

Supplementary Table S1

Actin gene accession #	Primer direction	Primer sequence 5’ – 3’	Amplicon (promoter) length, bp
XM_021049442.1	Forward	CCTCTTGGCAACGTAACACCAAC	1479
XM_021049442.1	Reverse	TTTGTCAGTAGTTTTGAATTGAGA	1479
XM_021045858.1	Forward	GGCATCCGTCTCGTCACGATAG	1504
XM_021045858.1	Reverse	TTTAATATATTTTAGAAAGGTCAAACACC	1504
XM_021060755.1	Forward	CTGACATCAGCCGTACTCTAGAAC	1463
XM_021060755.1	Reverse	TGTAAGTTTGAAGTATGAAGTGAA	1463
KXJ15097.1	Forward	TATATTGACGATCCAAGTGTCACA	998
KXJ15097.1	Reverse	GTTGGTTYTAAAATTTACTTACAACGAACA	998
